# Invasive *Cunninghamella bertholletiae* Infections in children: a case series and literature review

**DOI:** 10.3389/fped.2026.1847039

**Published:** 2026-05-28

**Authors:** Xue Li, Kunjiao Xue, Jiakai Li, Tingting Zhang, Xiaoxi Lu, Chuanjie Yuan, Jin Wu, Xiangfei Zeng, Min Shu, Juanjuan Lyu

**Affiliations:** 1 Department of Pediatric, West China Second University Hospital, Sichuan University, Chengdu, Sichuan, China; 2 Key Laboratory of Birth Defects and Related Diseases of Women and Children, Sichuan University, Ministry of Education, Chengdu, Sichuan, China; 3 Department of Pathology, West China Hospital, Sichuan University, Chengdu, Sichuan, China

**Keywords:** children, *Cunninghamella bertholletiae*, Isavuconazole, mNGS, mucormycosis

## Abstract

Pediatric infections caused by *Cunninghamella bertholletiae (C. bertholletiae)* are exceedingly rare with only few published case reports. Herein, we describe five cases of *C. bertholletiae* diagnosed and treated in Pediatric Hematology-Oncology Patients. These cases indicated that pediatric *C. bertholletiae* infection presents with non-specific clinical features, is associated with high fatality, and frequently involves multiple anatomical sites, most commonly the lungs, demonstrating epidemiological patterns distinct from those observed in adults. From a diagnostic perspective, mNGS enables rapid pathogen identification, facilitates timely initiation of antifungal therapy, and may improve survival. Therapeutically, surgical intervention is associated with a significant reduction in mortality. Although combination therapy and monotherapy appear to have comparable overall efficacy, sequential monotherapy demonstrates a more pronounced survival benefit. Furthermore, Isavuconazole represents an effective salvage option and is linked to a significant decrease in mortality.

## 1 Introduction

The *Cunninghamella* species are members of the order Mucorales (class *Zygomycetes*) and can induce rare but highly lethal infections in children. Among them, *Cunninghamella bertholletiae* (*C. bertholletiae*) is the most prevalent, accounting for up to 86.4% of *Cunninghamella* infections and approximately 6.3 to 8% of all mucormycosis cases (1). Clinically, cases are characterized by an abrupt onset and rapid progression, leading to a high fatality rate. *C. bertholletiaeit* *>* primarily affects immunocompromised hosts, with established risk factors including diabetes mellitus, hematopoietic stem cell transplantation (HSCT), severe trauma, burns, and hematological malignancies (2, 3). These infections occur in a vulnerable population, with an overall incidence of invasive fungal infections ranging from 4.9% to 7.2% and a mortality rate of 21.7% to 59% (4). The most severe presentation is disseminated mucormycosis, particularly with central nervous system (CNS) involvement, which is associated with a mortality rate of approximately 80% (5). Of these, *C. bertholletiae* carries the highest fatality risk compared with other *Cunninghamellait* *>* species (1).

Despite increasing recognition, pediatric *C. bertholletiae* infection remains infrequently reported worldwide, and its clinical profile is still incompletely characterized. Herein, we describe five cases diagnosed in Western China in 2025. An integrated analysis of these cases and the existing literature was conducted to elucidate the clinical presentation, diagnostic approaches, and management of this disease in the pediatric population, with the aim of improving clinical understanding.

## 2 Case Report

### 2.1 Case 1

A 13-year-old girl was diagnosed with precursor B-cell acute lymphoblastic leukemia (pre-B ALL) and initiated induction chemotherapy. On day 9 following chemotherapy initiation, she developed recurrent high fever and abdominal pain. Despite empirical antimicrobial therapy with vancomycin, meropenem, Voriconazole (VORI), and Trimethoprim-Sulfamethoxazole (TMP-SMX), her symptoms failed to improve. On day 14, blood metagenomic next-generation sequencing (mNGS) detected *C. bertholletiae* (sequence count: 46; high confidence). Urine mNGS also identified *C. bertholletiae* (sequence count: 48; high confidence), while the (1,3)-β-D-glucan (BG) and galactomannan (GM) tests were negative. Contrast-enhanced computed tomography (CT) of the chest and abdomen demonstrated involvement of the lungs, kidneys, pancreas, and spleen. At that time, the absolute neutrophil count (ANC) had remained below 500/mm^3^ for 10 days. She was therefore clinically diagnosed with disseminated mucormycosis. VORI was discontinued, and treatment was initiated with Amphotericin B (AmB) at a dose of 0.5–1 mg/kg/day combined with posaconazole (PSZ) at 300 mg/day. On day 16, the patient became afebrile, and her abdominal pain gradually resolved. On day 17, ANC recovered to above 500/mm^3^ with concomitant granulocyte colony-stimulating factor (G-CSF) support. The patient ultimately achieved full recovery.

### 2.2 Case 2

A 4-year-old boy was diagnosed with acute myeloid leukemia (AML), M4 subtype, classified as high risk. He underwent myeloablative, major ABO-compatible, HLA-haploidentical HSCT from his father in January 2025. Post-transplant engraftment was achieved on day 9 for platelets and on day 11 for neutrophils. On day 145, he developed pancytopenia, with ANC persistently below 500/mm^3^, requiring repeated red blood cell and platelet transfusions. On day 154, the patient developed recurrent fever, and blood mNGS detected *Enterococcus faecium*. Treatment with linezolid, meropenem, Micafungin (MCFG), and TMP-SMX was initiated, and he became afebrile by day 160. However, on day 170, the fever recurred. Despite adjustment of the antimicrobial regimen to polymyxin B, tigecycline, and voriconazole, the fever persisted. Blood mNGS on day 173 detected *C. bertholletiae* (sequence count: 120; high confidence), while BG and GM tests remained negative. Chest CT revealed pulmonary involvement (Figure 1). The patient was clinically diagnosed with sepsis complicated by pulmonary mucormycosis. VORI was discontinued, and therapy with L-AmB (5 mg/kg/day) was initiated. On day 176, the patient developed dyspnea, cyanosis, vomiting, and progressive headache with drowsiness, consistent with disseminated mucormycosis complicated by acute intracranial hypertension. His condition further deteriorated on day 178, manifesting as central respiratory failure and septic shock. Endotracheal intubation was performed, and he was transferred to the Pediatric Intensive Care Unit (PICU). Therapeutic hypothermia was initiated for neuroprotection, which precluded accurate core temperature monitoring. Owing to the patient's critical and hemodynamically unstable condition, lumbar puncture and contrast-enhanced CT could not be performed. On day 179, care was withdrawn at the family's request, and the patient died. Throughout this period, ANC remained persistently below 500/mm^3^.

**Figure 1:**
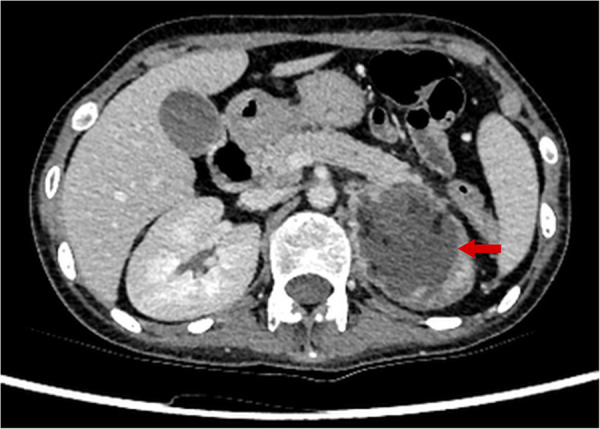
Abdominal computed tomography (CT) revealed a hypodense lesion in the upper pole of the left kidney.

### 2.3 Case 3

An 11-year-old boy was diagnosed with B-ALL, L2 subtype, and started chemotherapy according to the CCCG-ALL-2020-I/HR protocol. On day 7 post-chemotherapy, ANC dropped below 500/mm^3^. By day 9, he developed a fever and cough. Despite treatment with meropenem, vancomycin, and VORI, the fever persisted. Chest and abdominal CT revealed pulmonary involvement (Figure 2). Blood mNGS performed on day 12 detected *C. bertholletiae* (sequence count: 4, moderate confidence), while BG and GM tests were negative. Pulmonary mucormycosis was suspected, and treatment with AmB (0.5–1 mg/kg/day for 15 days) was initiated. By day 14, he became afebrile, and by day 15, his ANC had recovered above 500/mm^3^ with support from G-CSF therapy. He was subsequently transitioned to sequential therapy with posaconazole (300 mg/day). The patient ultimately made a full recovery.

**Figure 2:**
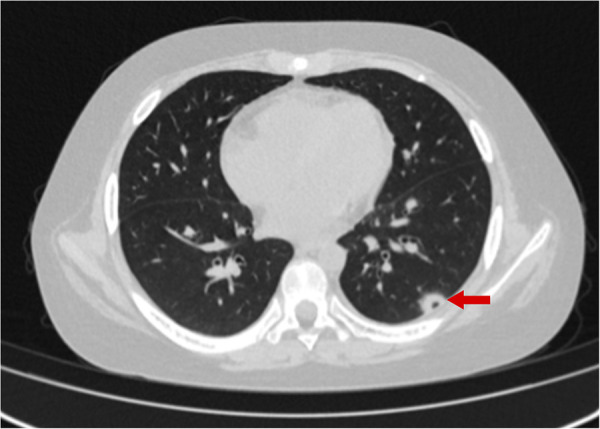
Chest computed tomography (CT) showing a 1.4 × 0.8 cm nodule with a small central cavitation.

### 2.4 Case 4

A 13-year-old boy was diagnosed with EBV-associated hemophagocytic lymphohistiocytosis (HLH) and treated according to the 1994 HLH protocol. On day 7 post-chemotherapy, he developed recurrent fever, cough, and abdominal pain, with an ANC below 500/mm^3^. Despite treatment with meropenem, linezolid, MCFG, and ganciclovir, the fever persisted. Contrast-enhanced CT revealed involvement of the lungs, spleen, and pancreas (Figure 3), while brain MRI indicated intracranial involvement. On day 15, blood mNGS detected *C. bertholletiae* (sequence count: 517, high confidence), while BG and GM tests remained negative. The patient was clinically diagnosed with disseminated mucormycosis. Treatment with L-AmB (10 mg/kg/day) in combination with Isavuconazole (ISZ) at 200 mg/day was initiated. By day 19, he became afebrile, and his ANC had recovered above 500/mm^3^ with G-CSF therapy. Due to economic considerations, the regimen was subsequently adjusted to conventional AmB (1 mg/kg/day) combined with ISZ (200 mg/day) for 20 days. However, due to recurrent severe hypokalemia, the treatment was switched back to L-AmB (10 mg/kg/day) combined with ISZ (200 mg/day). On day 25, the patient developed ear discharge. Otoendoscopy revealed a brownish discoloration of the right tympanic membrane, raising suspicion for fungal otitis media. Maxillofacial magnetic resonance imaging (MRI) indicated involvement of the parotid gland, and Fluconazole (FLUCO) otic drops were administered. On day 45, the child developed masses on the right lower extremity. MRI revealed abnormal signal intensity in the right sartorius muscle (Figure 4). On day 55, the patient was discharged at the family's request due to economic considerations. Sequential therapy with ISZ (200 mg/day) was continued for two months post-discharge before being self-discontinued. The patient is alive.

**Figure 3:**
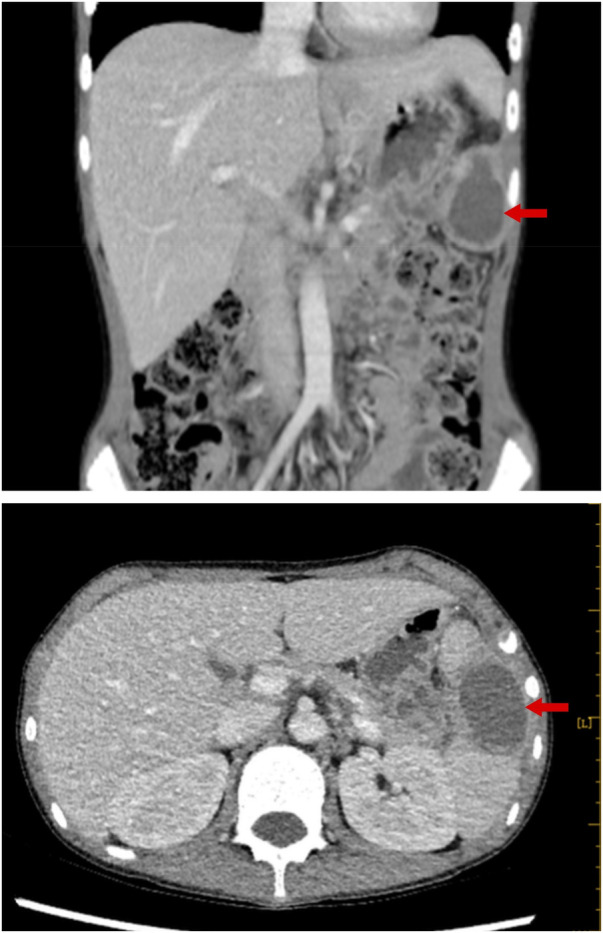
Abdominal computed tomography (CT) showing irregular hypodense lesions in the spleen, measuring 4.1 × 4.7 × 2.9 cm.

**Figure 4:**
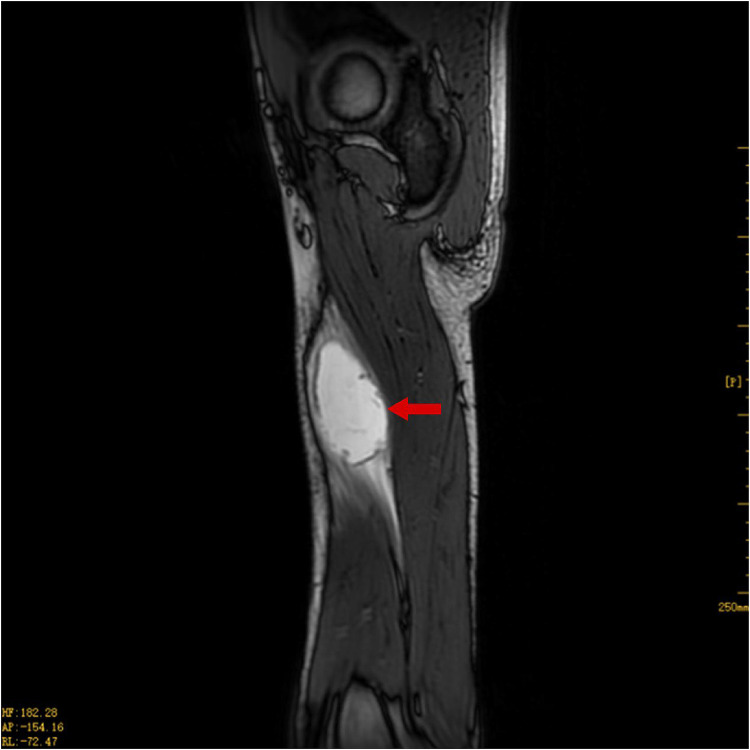
Magnetic resonance imaging (MRI) showing a well-defined cystic lesion within the right sartorius muscle, demonstrating T2 hyperintensity and measuring 7.2 × 3.8 × 3.3 cm.

### 2.5 Case 5

A 12-year-old boy was diagnosed with very severe aplastic anemia (vSAA). Following the diagnosis, he developed persistent pancytopenia, with an ANC continuously below 500/mm^3^, and required repeated transfusions of packed red blood cells and platelets. On day 21 post-diagnosis, he developed recurrent fever and chest pain. Despite treatment with vancomycin, meropenem, VORI and TMP-SMX, the fever persisted. Chest CT revealed pulmonary involvement. On day 25, blood mNGS detected *C. bertholletiae* (sequence count: 198, high confidence), leading to a clinical diagnosis of pulmonary mucormycosis. Treatment with L-AmB (5 mg/kg/day) combined with PSZ (300 mg/day) was initiated, and the antimicrobial regimen was adjusted to linezolid, meropenem, and acyclovir. On day 29, the patient became afebrile but continued to experience significant chest pain, with an ANC persistently below 500/mm^3^. A left upper lobectomy was performed on September 30, 2025. Pathological examination confirmed fungal infection (Figure 5). Fever recurred three days postoperatively (day 36), and again on day 47. Blood mNGS continued to detect *C. bertholletiae* (sequence count: 13,197, high confidence). The current treatment regimen was continued, but the fever persisted. Following a multidisciplinary team (MDT) discussion and thorough communication of the risks with the family, a decision was made to proceed with HSCT as rescue therapy. The plan was to perform a myeloablative, major ABO-compatible, HLA-haploidentical HSCT from his sister. However, one day prior to HSCT intervention, he developed dyspnea and septic shock, necessitating transfer to the PICU for intensive management. Despite resuscitative efforts, refractory hypotension persisted, followed by sudden cardiac arrest. Care was withdrawn per the family's request, and the patient subsequently died. During this period, his ANC remained below 500/mm^3^, accompanied by persistent fever.

**Figure 5:**
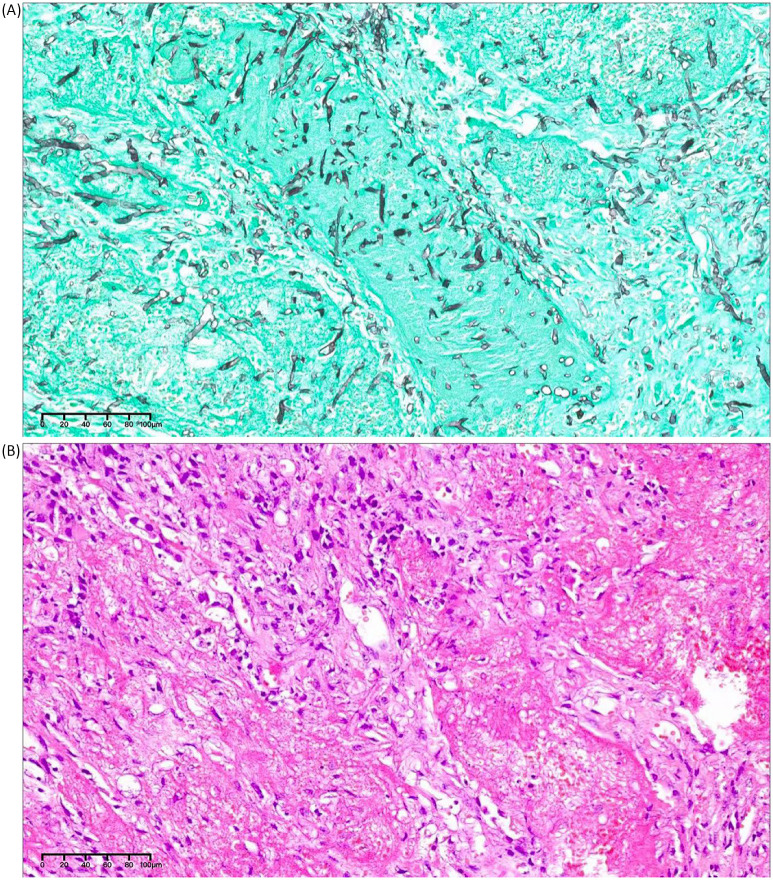
Microscopic images of lung tissue samples exhibiting features of mucormycosis. **(A)** Grocott's methenamine silver (GMS) stain showing broad, pauciseptate, irregularly branching hyphae (Objective 40×). **(B)** Hematoxylin and eosin (H&E) stain showing broad, ribbon-like, aseptate hyphae (Objective 40×).

## 3 Discussion

Among the causative agents of mucormycosis, the *Rhizopus* species are the most common, followed by *Mucor* and *Cunninghamella* species*.* Within the *Cunninghamella* genus, *C. bertholletiae* is the most frequently reported species. Although *C. bertholletiae* accounts for a minority of mucormycosis cases, it represents an emerging cause of disseminated disease (6). This infection primarily occurs in immunocompromised hosts and is associated with a significantly higher mortality rate compared to other Mucorales pathogens (3).

A literature search identified 26 reported cases of *C. bertholletiae* infection in children (Table 1). The present study adds five additional cases, bringing the total to 31. The mean age of the patients was 11.19 ± 4.54 years. All 31 cases had underlying conditions, with hematological malignancies being the most common (25/31, 80.6%), followed by severe non-neoplastic hematologic disorders (4/31, 12.9%). Trauma and liver disease each accounted for one case. In contrast to adults, where diabetes mellitus is the most frequent underlying condition for mucormycosis, hematological malignancies predominate in children. No cases of diabetes were identified as an underlying condition in the pediatric cohort of this study. In contrast to prior reports, severe non-neoplastic hematologic disorders emerged as the second most common underlying condition in this series. This finding may be explained by the frequent presence of additional risk factors in these patients, such as neutropenia or treatment with the iron-chelating agent deferoxamine.

**Table 1:** Cases of pediatric *C. bertholletiae* infections.

Case/reference	Age, Years/sex	Underlying condition	Involved Organs	Coinfection	Previous Antifungal use	Diagnostic method	Diagnosis	Antifungals	Surgery	ANE at the time of diagnosis (/mm^3^)	Duration of ANE <500/mm^3^ before diagnosis	Recovery time of ANE after treatment	Outcomes
Case 1	13/F	ALL-Pre B	Lungs, kidneys, pancreas, and spleen	*Bacteroides vulgatus, JC polyomavirus*	VORI, TMP-SMX	CT, mNGS	Disseminated mucormycosis	AmB with PSZ	No	30	10d	3d	Survived
Case 2	4/M	AML-M4, HSCT, GVHD	Lungs, CNS	*CMV, BKV, HHV-7, Enterococcus faecium, Klebsiella pneumoniae*	VORI, TMP-SMX, MCFG	CT, mNGS	Disseminated mucormycosis	L-AmB	No	0	28d	remained below 500/mm^3^	Deceased 3 days after diagnosis
Case 3	11/M	ALL-L2	lungs	No	VORI,TMP-SMX	CT, mNGS	Pulmonary mucormycosis	AmB, Sequential therapy with PSZ	No	120	5d	1d	Survived
Case 4	13/M	HLH	Lungs, spleen, pancreas, the parotid gland, aural region, CNS	EBV, tuberculosis	VORI, TMP-SMX	CT, MRI, mNGS	Disseminated mucormycosis	L-AmB × 4d, AmB × 20d, L-AmB × 14d, along with ISZ, Sequential therapy with ISZ for two months	Yes	470	7d	4d	Survived
Case 5	11/M	vSAA, HSCT	Lungs	*Pseudomonas aeruginosa, Granulicatella adiacens, HSV-1, Staphylococcus aureus,*	VORI, TMP-SMX	CT, mNGS, HPE	Pulmonary mucormycosis	L-AmB along with PSZ	Yes	0	28d	remained below 500/mm^3^	Deceased 1 day prior to HSCT
(16)	13/M	Cirrhosis, chronic glomerulonephritis	Heart, spleen, kidney	*Aspergillus*	No	NA	Disseminated mucormycosis	No	No	NA	NA	NA	Deceased
(17)	15/M	ALL	Lungs	NA	No	CT, HPE and culture	Pulmonary mucormycosis	L-AmB	Yes	<500	NA	NA	Survived
(18)	17/M	ALL, HSCT, GVHD	lungs, CNS	No	VORI	mNGS, MRI	Disseminated mucormycosis	L-AmB along with PSZ	Yes	NA	NA	NA	Survived
(19)	5/M	Mature B-ALL	Lungs, musculoskeletal, skin	No	AmB, VORI and CAS	CT, PCR	Disseminated mucormycosis	L-AmB, along with TER and oral PSZ	Yes	0	NA	NA	Survived
(20)	15/M	Relapsed AML	Lungs, paranasal sinus, orbit	NA	FLUCO	CT, HPE and culture	Disseminated mucormycosis	AmB × 3d, then L-AmB	No	0	NA	remained below 500/mm^3^	Deceased
(21)	3/M	AML	Lungs	No	AmB [1 mg/(kg • d)]	CT, Fungal culture	Pulmonary mucormycosis	AmB	Yes	0	NA	50d	Deceased
(21)	7/M	Acute undifferentiated leukemia	Lungs	No	AmB [1 mg/(kg • d)]	Chest roentgenography, Fungal culture	Pulmonary mucormycosis	AmB	Yes	0	NA	remained below 500/mm^3^	Deceased
(4)	13/F	ALL	Lungs, spleen, CNS, skin	NA	VORI, TMP-SMX	CT, HPE and PCR	Disseminated mucormycosis	L-AmB	Yes	<500	NA	NA	Deceased
(2)	7/M	major *β*-thalassemia	Cardiovascular system, Lungs	*COVID-19*	No	CT, PCR analysis	Disseminated mucormycosis	No	Yes	NA	NA	NA	Deceased
(22)	14/F	ALL	Lungs	NA	No	HPE and culture	Pulmonary mucormycosis	L-AmB × 30d, Sequential therapy with PSZ 14 months	Yes	<500	7d	NA	Survived
(23)	4/M	ALL	Lungs	NA	MCFG	CT, PCR	Pulmonary mucormycosis	L-AmB	No	<500	NA	NA	Deceased
(24)	17/F	Relapsed ALL	Lungs	NA	AmB	CT, culture	Pulmonary mucormycosis	L-AmB × 40d, Sequential therapy with PSZ	No	<500	NA	NA	Survived
(25)	7/M	Trauma	Skin	*A. flavus, Staphylococcus aureus*	NA	Culture, molecular histopathology	Cutaneous mucormycosis	L-AmB, Sequential therapy with ISZ	Yes	NA	NA	NA	Survived
(26)	7/F	ALL	Lungs	NA	VORI	CT, HPE and PCR analysis	Pulmonary mucormycosis	L-AmB, CAS along with ISZ	Yes	<500	NA	NA	Survived
(27)	8/M	Relapsed lymphosarcoma	Larynx, lungs, heart, esophagus, stomach, ileum	*Candida*	Mycostatin	Autopsy	Disseminated mucormycosis	No	No	NA	NA	NA	Deceased
(28)	17/F	Relapsed ALL	Lungs	NA	No	Chest x-ray, HPE	Pulmonary mucormycosis	L-AmB	Yes	NA	NA	NA	Survived
(29)	15/M	AML, HSCT, GVHD	Lungs	*CMV*	VORI	Fungal culture, CT	Pulmonary mucormycosis	AmB	No	NA	NA	NA	Deceased
(30)	10/M	ALL-B	Lungs	NA	No	Chest radiography, Culture	Pulmonary mucormycosis	L-AmB	No	<500	NA	remained below 500/mm^3^	Deceased
(31)	10/F	Second relapsed AML, HSCT, GVHD	Lungs	*EBV, CMV, Pneumocystis jiroveci*	VORI	CT, HPE and fungal culture	Pulmonary mucormycosis	L-AmB, Sequential therapy with PSZ	Yes	NA	NA	NA	Deceased
(32)	16/F	Ganglioneuroma, multivisceral transplant, GVHD	Skin	*C. albicans, C. glabrata, Cryptosporidium*	FLUCO, CAS	culture	Cutaneous with deep tissue involvement	AmB × 2d, L-AmB × 7d, Sequential therapy with osaconazole	Yes	800	NA	NA	Survived
(33)	5/F	Anaplastic stage III Wilms tumor	Lungs, skin, CNS, kidney	NA	MCFG	CT, HPE, culture	Disseminated mucormycosis	L-AmB	Yes	>500	5d	-	Survived
(34)	16/F	Pre B-ALL	Lungs	NA	TMP-SMX	CT, HPE and culture	Pulmonary mucormycosis	AmB, then L-AmB	Yes	0	NA	NA	Survived
(5)	8/M	β-thalassemia, HSCT	Cardiovascular system	*HHP-6B, human polyomavirus 4*	PSZ, CAS	CT, mNGS	Disseminated mucormycosis	AmB × 41d, L-AmB × 5d, along with PSZ, isavuconazonium	No	<500	NA	NA	Deceased +56d after HSCT
(35)	17/NA	ALL-B, HSCT, GVHD	Lungs, skin,	No	LAmB, TMP-SMX	CT, culture and pan-fungal PCR	Disseminated mucormycosis	L-AmB along with TER and PSZ	Yes	NA	NA	NA	Deceased
(19)	17/F	Relapsed ALL, HSCT	Skin, lungs	*C. albicans*	VORI, L-AmB	CT, The panfungal PCR and culture	Disseminated mucormycosis	L-AmB, along with TER and oral PSZ	Yes	>500	NA	NA	Deceased
(36)	7/F	sVAA, HSCT	Lungs	No	L-AmB	CT, NGS	Pulmonary mucormycosis	L-AmB, along with ISZ	Yes	NA	NA	NA	Survived

M, male; F, Female.

The overall case-fatality rate of mucormycosis ranges from 40% to 80%, depending on host comorbidities and the anatomical site of infection (3). Disseminated infection, particularly involving the CNS, carries a mortality rate exceeding 80% (3, 5). Studies have previously identified disseminated infection and HSCT as two major independent risk factors for mortality in pediatric mucormycosis (7). Herein, the overall mortality rate was 51.6% (16/31). Among the 14 cases of disseminated mucormycosis, 9 patients (64.3%) died. Ten patients (31.25%) had undergone bone marrow or solid organ transplantation, of whom 7 (70%) died. Consistent with prior research, disseminated infection and transplantation represent high-risk factors for mucormycosis and are associated with increased mortality. At the time of diagnosis, 19 patients had an ANC below 500/mm^3^, and 10 of these patients (52.6%) died. Notably, all three patients whose ANC remained below 500/mm^3^ died (100%), indicating that profound and prolonged neutropenia is a significant risk factor for mortality. Analysis of the 31 cases also revealed that co-infections, present in 13 patients (41.9%), were associated with a substantially higher mortality rate (69.2%) compared with monomicrobial *C. bertholletiaeit* *>* infections (42.9%, 7 cases). This finding indicates that co-infection is an important risk factor for death.

The most common clinical manifestations of mucormycosis in children include rhino-orbitocerebral, pulmonary, cutaneous, and gastrointestinal involvement. Previous studies have reported that rhino-orbitocerebral mucormycosis typically occurs in patients with diabetes (3, 6). Here, the most frequently involved sites were the lungs (27/31), skin (7/31), and CNS (5/31). These findings suggest that, in pediatric *C. bertholletiaeit* *>* infections, the incidence of rhino-orbitocerebral mucormycosis may have been overestimated, while pulmonary mucormycosis may have been underestimated. No cases of gastrointestinal involvement were identified. Clinical manifestations vary by site, with recurrent high fever refractory to antibiotic therapy being the most common presenting feature. In the present study, among the 14 cases of disseminated mucormycosis, 9 patients (64.3%) died. Of the 15 cases with pulmonary mucormycosis, 7 patients (46.7%) died, whereas neither of the two patients with cutaneous mucormycosis died. These findings suggest that children are more prone to multi-system involvement, resulting in disseminated mucormycosis, which is associated with a high mortality rate. Among the 27 cases with pulmonary involvement, chest imaging studies revealed findings consistent with fungal infection.

Currently, the diagnosis of mucormycosis relies on mycological culture and histopathological evaluation (3, 6). A major diagnostic challenge is the limited sensitivity of culture, which is positive in only about half of cases with microscopy-confirmed hyphae, thereby increasing the risk of delayed or missed diagnosis. Unlike some other fungal infections, mucormycosis cannot be detected using BG or GM tests (8, 9). In our center, all five pediatric patients tested negative for both BG and GM. Notably, a diagnostic delay of six days is associated with a sharp increase in 30-day mortality, rising from 35% to 66%. These findings reflect the critical need to develop rapid and accurate methods for early detection (10). Studies have reported that serum PCR testing for mucormycosis can achieve a sensitivity of 85.2% and a specificity of 89.8% (11). As a modern molecular biology technique, mNGS represents a significant advancement in clinical diagnostics. With the ability to detect over 15,000 pathogen species with known genomic sequences, coupled with high sensitivity, rapid turnaround time, and the capacity to identify rare infections, mNGS substantially improves pathogen detection rates in clinical practice (12, 13).

In the present study, 13 cases were confirmed by histopathological examination (HPE) or culture, with 7 deaths, corresponding to a mortality rate of 53.8%. Among 7 cases diagnosed by PCR, 5 resulted in death, yielding a mortality rate of 71%, notably higher than previously reported, though the underlying reasons remain unclear. In contrast, of the 8 cases identified via mNGS, only 2 patients died, corresponding to a mortality rate of 25%. These findings indicate that mNGS could reduce both the time to definitive diagnosis and associated mortality, suggesting its potential as a robust method for early detection of mucormycosis.

Current guidelines advocate early and comprehensive surgical intervention for mucormycosis whenever feasible (3). For first-line pharmacologic treatment, the preferred agent is L-AmB at 5–10 mg/kg/day. ISZ is an alternative, with PSZ also an option. The ISZ regimen consists of an intravenous loading dose of 10 mg/kg (maximum 200 mg per dose) every 8 hours for 2 days, followed by a maintenance dose of the same amount once daily. The duration of therapy is individualized and depends on two criteria: permanent resolution of immunosuppression and achievement of a complete radiographic response. In the setting of salvage therapy, ISZ is given the highest priority, with PSZ as an alternative and L-AmB as another potential option (14, 15).

Among the patients, 20 underwent surgical intervention, with 8 deaths (mortality rate of 40%). Conversely, among the 11 patients who did not undergo surgery, 8 died (72.7%). These findings indicate that surgical intervention significantly reduces mortality. Moreover, all 3 patients who did not receive antifungal therapy died. Notably, all 4 patients treated with ISZ survived. A previous study (14) reported a success rate of 78.6% for ISZ in children, while our data, showed survival in the only pediatric patient treated with this agent, further supporting its marked effectiveness against mucormycosis in this population. Proactive use of ISZ in treatment is therefore recommended to reduce mortality.

Among patients receiving combination therapy (*nit* *>*  = 10), the overall mortality was 40% (4/10). Mortality was high with PSZ combinations (4/7), but none were reported with ISZ combinations (0/3). Among those receiving monotherapy (*n* = 18), the mortality rate was 50% (9/18), with high mortality observed for AmB/L-AmB alone (8/12) but only one death among patients receiving sequential therapy (1/6). Although both therapeutic strategies are valid options, for combination therapy, clinicians should prioritize ISZ in combination with AmB/L-AmB. If monotherapy is used, a sequential regimen incorporating a follow-on azole is strongly recommended to optimize outcomes. For pediatric patients who are critically ill with rapidly progressive disease, ISZ remains the preferred salvage agent.

In conclusion, *C. bertholletiae* infection in children is characterized by nonspecific symptoms, frequent pulmonary involvement, and high mortality. Its epidemiology, including predisposing conditions and infection sites, differs from that observed in adults. The limited sensitivity of conventional diagnostics can delay treatment, whereas mNGS improves detection rates, accelerates diagnosis, and is associated with lower mortality. Surgical intervention significantly reduces mortality and should be performed promptly upon diagnosis. Both monotherapy and combination therapy are valid treatment strategies; however, sequential regimens in monotherapy are associated with notably lower mortality. ISZ, when used as salvage therapy, substantially reduces mortality and warrants early initiation. Nevertheless, further research is required to refine diagnostic and therapeutic approaches, as early detection and timely intervention are critical to improving outcomes in pediatric mucormycosis.

## Data Availability

The raw data supporting the conclusions of this article will be made available by the authors, without undue reservation.
